# Growth and superconductivity of niobium titanium alloy thin films on strontium titanate (001) single-crystal substrates for superconducting joints

**DOI:** 10.1038/s41598-018-33442-7

**Published:** 2018-10-11

**Authors:** Yuhei Shimizu, Kazuhiko Tonooka, Yoshiyuki Yoshida, Mitsuho Furuse, Hiroshi Takashima

**Affiliations:** 1 National Institute of Advanced Industrial Science and Technology (AIST), Central 2, 1-1-1 Umezono, Tsukuba, Ibaraki 305-8568 Japan; 2 Present Address: National Metrology Institute of Japan (NMIJ), National Institute of Advanced Industrial Science and Technology (AIST), Central 3, 1-1-1 Umezono, Tsukuba, Ibaraki 305-8563 Japan

## Abstract

Aiming to introduce NbTi alloy superconducting joints for REBa_2_Cu_3_O_7−δ_ (REBCO, RE: rare-earth element) superconducting wires, NbTi alloy thin films were deposited at room temperature on SrTiO_3_ (STO) (001) single-crystal substrates, which have a high lattice matching with REBCO (001). The strain, crystallinity, surface morphology, and superconducting property of the films with various thicknesses were investigated. The NbTi films grew in the orientation with (110)NbTi//(001)STO:[001]NbTi and [11–0] NbTi//[100]STO; that is, the NbTi lattices had two directions in the (110) of NbTi. The strain decreased and the crystallinity improved as the film thickness increased. The films were found to crystallize immediately at the interface between the films and substrates by cross-sectional scanning transmission electron microscopy. The flat surfaces of the films have mesh-like morphologies due to the growth of elongated NbTi grains along the [100] and [010] of the STO, reflecting the in-plane two directions of the NbTi lattices. The superconducting transition temperature of the films increased with improvement in the crystallinity of the films. The preparation of superconducting NbTi alloy thin films with sufficient crystallinity at room temperature suggested the possibility of forming the films on REBCO and the applicability of the films as superconducting joints.

## Introduction

It has been studied for practical application of REBa_2_Cu_3_O_7−δ_ (REBCO, RE: rare-earth elements) high-temperature superconductors which are usable at liquid nitrogen temperature^1,2^. Recently, the REBCO high-temperature superconductors have been expected to be used in high-magnetic-field magnetic resonance imaging (MRI) equipment^3–5^. One of the technologies required for high-magnetic-field MRI is the joint technique of REBCO-coated conductors (CCs)^6^. In the direct joint method connecting REBCO superconducting layers in the REBCO CCs, the joint structure is simple. However, a complicated joining technique and long processing time are required because the superconducting properties of REBCO are strongly affected by the directions of REBCO crystals and the oxygen content in REBCO^6–8^. In contrast, the indirect joint method connecting the REBCO layers in the CCs via another material can avoid the problems of the direct joint method. Joints of Ag or Cu layers on the REBCO layers in CCs are one of the indirect joints^9–12^. However, a large joint area is necessary for a low-resistance circuit because Ag and Cu are not superconductors, although they have low resistivities (0.2–0.3 × 10^−8^ Ωm at liquid nitrogen temperature)^13^. In order to achieve low resistance varying from 10^−13^ Ω to 10^−14^ Ω, which is required for operating MRI in a persistent current mode^6^, in the case of a Ag layer having a thickness of 10 μm and width of 5 mm, the joint area is calculated to range from 0.6 m^2^ to 6 m^2^, i.e., the joint length ranges from 120 m to 1200 m at liquid nitrogen temperature. Therefore, in the indirect joint method, the joint material should be a superconductor.

A NbTi alloy is a superconductor with wide applications including in current MRI equipment^5,14^. There are simple superconducting joint techniques that can be used with the NbTi alloy^15,16^. Although the superconducting transition temperature (*T*_*c*_) of NbTi alloy is lower than that of REBCO, indirect joints via NbTi alloy layers stacked on REBCO layers are more advantageous than the direct joints of REBCO layers in terms of easier superconducting joint technique. We have surveyed the literature on the potential application of superconducting joints of NbTi alloy; however, little data is available on NbTi alloy thin films. The properties of NbTi alloy thin films formed on REBCO, such as their strain, crystallinity, morphology, and superconductivity have not been understood in detail. When investigating the properties of NbTi alloy thin films deposited on REBCO films or tapes, the analysis becomes complicated because not only the NbTi alloy thin films but also the REBCO are influencing parameters. Therefore, in the present study, NbTi alloy thin films were deposited on SrTiO_3_ (STO) (001) single-crystal substrates as a preliminary research before depositing them on REBCO in order to investigate the properties of NbTi alloy thin films. STO is a typical cubic perovskite-type oxide with lattice parameter *a* = 3.901 Å, and is chemically stable^17^. Orthorhombic YBa_2_Cu_3_O_7−δ_ (YBCO), as a typical REBCO, has a perovskite-unit. YBCO is generally oriented to the [001] direction on CC substrates^18^. Lattice parameters *a* and *b* constituting the YBCO (001) lattice are 3.8824 Å and 3.8282 Å, respectively^19^. The STO (001) lattice has high lattice matching with the YBCO (001) lattice with an average lattice mismatch of 1.2%. The strain, crystallinity, surface morphology, and superconducting transition of NbTi alloy thin films on STO (001) single-crystal substrates were investigated with varying film thickness as a parameter. Temperature is an important condition for prospective deposition on REBCO because the superconducting properties of REBCO deteriorate due to oxygen deficiency of REBCO at high temperature^6–8^. In addition, high temperature in film preparation could lead to a change of film composition. The film preparation without heating is expected to avoid the deterioration of REBCO and the change of composition of NbTi alloy thin films. Therefore, deposition of NbTi alloy films on STO was conducted at room temperature.

## Results and Discussion

**Figure 1:**
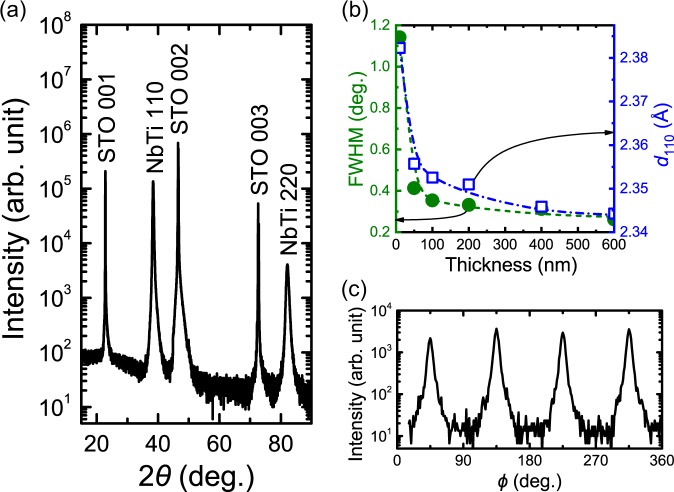
(**a**) θ–2θ scan XRD pattern of NbTi alloy thin film with 400 nm thickness on a STO substrate. (**b**) FWHM of 110 peaks of NbTi and NbTi (110) interplanar spacing (*d*_110_) as a function of thickness. The FWHMs were obtained from the intensity expressed on a linear scale. The *d*_110_ values were calculated from diffraction angles of the NbTi 220 peaks. (**c**) φ scan XRD pattern of NbTi alloy thin film with 400 nm thickness on a STO substrate. XRD peaks were detected every 90°.

Figure 1(a) shows the θ–2θ scan XRD pattern of a NbTi alloy thin film with a typical thickness of approximately 400 nm. In addition to the 00 *l* peaks derived from the STO (001) single crystal substrate, only intense peaks attributed to *hh*0 of the body-centered cubic NbTi alloy were observed. The NbTi alloy thin film crystallized at room temperature and was oriented to (110)NbTi//(001)STO. The NbTi alloy thin films grew in the [110] direction regardless of the film thickness because the NbTi peaks observed in all the thin films with thicknesses from 10 nm to 600 nm were attributable to only *hh*0. Figure 1(b) shows the full widths at half maximum (FWHMs) of the NbTi 110 peaks of the thin films and the NbTi (110) interplanar spacing (*d*_110_) calculated from the diffraction angles of the NbTi 220 peaks as a function of film thicknesses. The FWHMs were obtained from the intensity expressed on a linear scale. The FWHM narrowed with increasing film thickness, and especially sharply changed from 10 nm to 50 nm. The FWHM is influenced by the strain of NbTi lattice and the crystallite size (perpendicular to the surface) of NbTi. The decrease in the FWHM indicates the decrease in the strain and the increase in the crystallite size. Therefore, remarkable decrease in the strain and drastic crystal growth are deduced in the thickness range from 10 nm to 50 nm. The *d*_110_ values of all the thin films were larger than that of bulk NbTi (2.324 Å)^20^ and decreased with increasing thickness. The trend of change in *d*_110_ value was similar to that of the FWHM. Figure 1(c) shows the φ scan XRD pattern of a NbTi alloy thin film with a typical thickness of approximately 400 nm. The 200 diffraction peak of the NbTi alloy thin film grown in the [110] direction can be detected by tilting the XRD sample stage by 45° and adjusting the θ–2θ and φ angles. The φ scan XRD pattern was obtained with 360° rotation of the sample stage as the tilt angle of the sample stage and the θ–2θ angle were fixed. The rotation axis of the sample stage perpendicularly intersects the (*hh*0) planes of the NbTi alloy thin film. The NbTi (200) has dyad symmetry around the rotation axis of the sample stage. Therefore, when all the NbTi lattices were in-plane oriented in the same direction, it was predicted that the two peaks attributing to NbTi 200 would be detected during the 360° rotation. However, in fact, four peaks were observed every 90°, indicating the presence of NbTi lattices with a different orientation rotated by 90° in the (*hh*0) planes.

**Figure 2:**
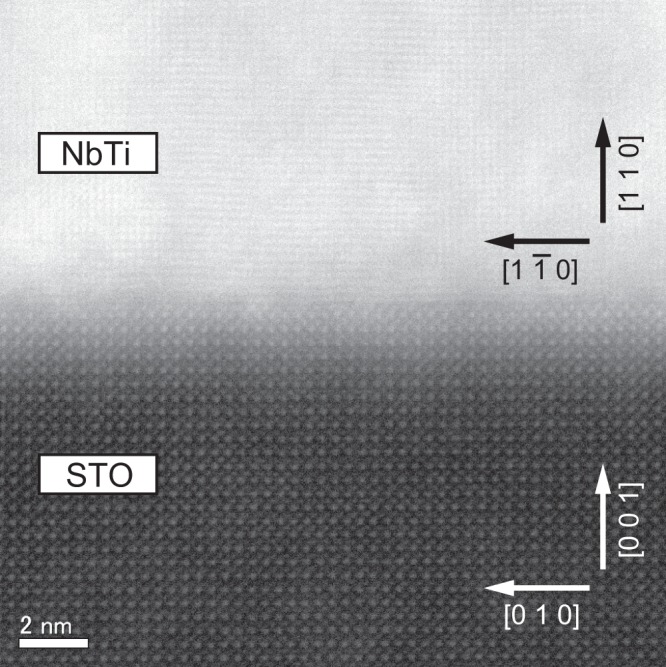
Cross-sectional STEM image of an interface between 400-nm-thick NbTi alloy thin film and STO substrate. The atomic image representing crystallized NbTi was observed at the interface.

The cross section of the interface between the STO (001) single-crystal substrate and the NbTi alloy thin film with a typical thickness of 400 nm is shown in the scanning transmission electron microscopy (STEM) image in Fig. 2. The view direction is along [100] of the STO. The vertical direction of the image is along [001] of the STO and [110] of the NbTi. In a dark field STEM image, the region with higher density appears brighter. The dark region in the lower half was attributed to the STO substrate and the bright region in the upper half was attributed to the NbTi alloy thin film. In the region of the STO substrate, an orderly arrayed atomic pattern was observed. In the NbTi alloy thin film region, the lattice fringe and dot patterns were observed even at the interface, indicating that a crystalline NbTi alloy thin film grew immediately on the STO substrate. Crystallization of the NbTi alloy thin film at the interface is an important result for achieving superconducting joints. Lateral spacing of the dot in the NbTi region was approximately 2.31 Å. Using the data of NbTi bulk^20^, it is derived that the dot spacings along $[1\bar{1}0]$ and [001] are 2.324 Å and 1.643 Å, respectively. Therefore, the lateral direction of the STEM image is along $[1\bar{1}0]$; that is, the view direction is along [001] of the NbTi. The shape of NbTi (110) surrounded by the unit cell is a 4.647 Å (along $[1\bar{1}0]$ of NbTi) × 3.286 Å (along [001] of NbTi) rectangle. On the other hand, the shape of STO (001) surrounded by the unit cell is a 3.901 Å × 3.901 Å square. Therefore, the NbTi alloy thin films on the STO substrates receive compressive stress along $[1\bar{1}0]$ and tensile stress along [001]. The magnitude of mismatches between NbTi and STO is 19.12% along $[1\bar{1}0]$ and 15.77% along [001]. The dot spacing along out-of-plane [110] of the NbTi was found to be approximately 2.35 Å from the STEM image. The dot spacing along [110] corresponds to the *d*_110_ value of NbTi. As shown in Fig. 1(b), the *d*_110_ value from the XRD was 2.346 Å, which was almost agree with the value obtained by the STEM image. The *d*_110_ value of the thin film was longer than that of the bulk value (2.324 Å)^20^. This is presumably due to the Poisson ratio tensile strain in out-of-plane [110], though it is not simple because of different stress along $[1\bar{1}0]$ and [001]. The difference ratio was 0.95% between the bulk *d*_110_ value and the thin film *d*_110_ value obtained from the XRD. The out-of-plane *a* axis parameter of the NbTi alloy thin film was indicated to be 3.29 Å from the values of dot spacing along $[1\bar{1}0]$ and [110] in the STEM image. From the 2θ angle of 200 peaks for XRD φ scan measurement, the *a* parameter was calculated to be 3.300 Å, which was close to the *a* from the STEM image. The *a* parameter of the thin film was also larger than the bulk value (3.286 Å)^20^. The difference ratio was 0.43% between the bulk *a* and the thin film *a* obtained from the XRD. The difference ratio of the *d*_110_ was larger than that of the *a*, indicating larger influence by the strain along [110]. The decrease in the *d*_110_ value with increasing film thickness, as shown in Fig. 1(b), strongly reflects the decrease in the strain due to the increase in the film thickness.

**Figure 3:**
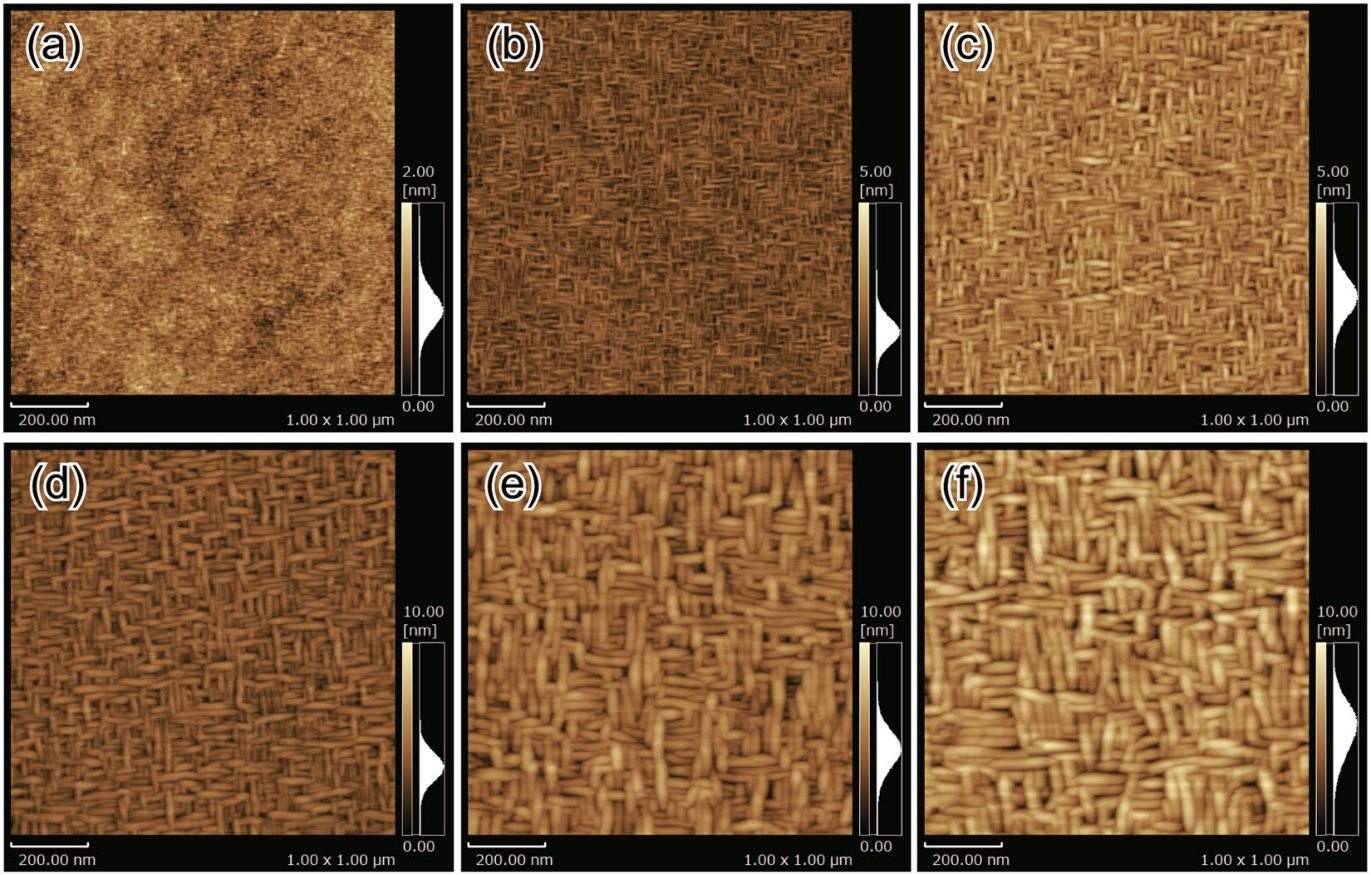
Surface AFM images of NbTi alloy thin films. The vertical and horizontal directions of the images are showed in accordance with [010] and [100] directions of STO (001) substrates, respectively. The film thicknesses are (**a**) 10 nm, (**b**) 50 nm, (**c**) 100 nm, (**d**) 200 nm, (**e**) 400 nm, and (**f**) 600 nm.

Figure 3(a–f) show the atomic force microscopy (AFM) images of the surfaces of NbTi alloy thin films with various thicknesses of 10, 50, 100, 200, 400, and 600 nm, respectively. The vertical and horizontal directions of the images are displayed in accordance with the [010] and [100] directions of the STO (001) substrate, respectively. For thin films with film thicknesses of 50 nm or more, elongated grains were observed and the surfaces appeared to be mesh-like, with the mesh directions along the [010] and [100] directions of the STO (001) substrate. It is conjectured that the surface morphology with orthogonal mesh directions originated from the growth of NbTi grains in two directions rotated by 90° in the NbTi (*hh*0) planes. Taking the rectangle shape of NbTi (110) into account, the long axis of the elongated grains is inferred to be along $[1\bar{1}0]$ of NbTi. The grains of the thin film with 10-nm thickness were so small that the mesh-like morphology could not be clearly understood. The NbTi grains grew with increasing film thickness, especially grew drastically from 10 nm to 50 nm. The case corresponds to the decreasing trend of the FWHM of the XRD peak. In the sample series of this study, both the strain and the crystallite size changed as the film thickness increased. According to the Williamson-Hall plot^21,22^, it is considered that the contribution of the strain is larger (see Supplementary Information). The increase in the film thickness resulted in the improvement in the crystallinity of the NbTi alloy thin films. The arithmetic average roughness (*R*_*a*_) of the NbTi alloy thin film surface was the lowest at 0.2 nm for the thin film with 10-nm thickness. The growth of NbTi grains with increasing film thickness led to increased surface roughness. Nevertheless, it is noteworthy that even the 600-nm-thick thin film with the largest *R*_*a*_ of 1.1 nm had a flat surface.

**Figure 4:**
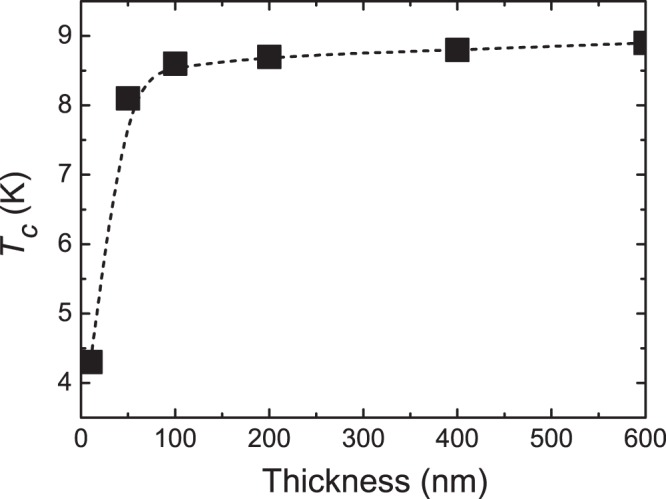
Film thickness dependence of *T*_*c*_ of the NbTi alloy thin film. The *T*_*c*_ is defined as the endpoint temperature of decrease in resistivity due to superconducting transition in the resistivity curve as a function of temperature. The *T*_*c*_ increased with thickness, and especially increased sharply from 10 nm to 50 nm.

Figure 4 shows the film thickness dependence of *T*_*c*_ of the NbTi alloy thin film. The *T*_*c*_ is defined as the endpoint temperature of decrease in resistivity due to superconducting transition in the resistivity curve as a function of temperature. Superconducting transition was observed in all the obtained thin films. Although the *T*_*c*_ of the thin films were lower than the bulk *T*_*c*_ (10.0 K)^23^, it was sufficient for use at liquid helium temperature of 4.2 K. The *T*_*c*_ of the thin films increased with thickness, and increased sharply from 10 nm to 50 nm. The increasing trend of *T*_*c*_ corresponded to the trend of the decrease in the strain and the increase in the crystallite size with increasing film thickness. Thus, it is concluded that the improvement in the superconducting properties of the NbTi alloy thin film was derived from the improvement in crystallinity.

## Conclusions

In this study, NbTi alloy thin films were formed on STO (001) single-crystal substrates at room temperature. The NbTi alloy thin film crystallized and oriented in the [110] of NbTi irrespective of the film thickness. The crystallinity of the films improved with increasing film thickness. The φ scan XRD pattern suggested the presence of NbTi lattice rotated by 90° in the NbTi (*hh*0) planes, and the cross-sectional STEM and surface AFM images supported the in-plane orientation of NbTi lattices. The cross-sectional STEM image also indicated that NbTi immediately crystallized at the interface with the substrate. At the thin film surfaces, elongated mesh-like morphologies were observed at a film thickness of 50 nm or more and exhibited surface flatness with an *R*_*a*_ of 1.1 nm or less. As the film thickness increased, the *T*_*c*_ of the thin films increased corresponding to the improvement in crystallinity. The formation of a superconducting NbTi alloy thin film on the STO (001) lattice, which highly matches with the REBCO (001) lattice, presents important results toward the formation of NbTi alloy thin films on REBCO and achieving superconducting joints. Consequently, we propose indirect joints using NbTi alloy thin films as a superconducting joint method at 4.2 K.

## Methods

### Sample preparation

The NbTi alloy thin films were deposited on an STO (001) single-crystal substrate by RF magnetron sputtering. A NbTi alloy (Nb:Ti = 50:50 at%) disk with a diameter of 5.08 cm and thickness of 2 mm was used as a sputtering target. The deposition chamber was evacuated to 10^−4^ Pa, and Ar gas was introduced into the chamber. During discharge, the pressure in the deposition chamber and the RF power were maintained at 1.0 Pa and 70 W, respectively, and the distance between the target and substrate was set at 25 mm. The substrate temperature was room temperature. Thin films with thicknesses ranging from 10 nm to 600 nm were obtained by controlling the duration of deposition of the thin films.

### Characterization

To evaluate the crystal phases of NbTi alloy thin films, X-ray diffraction (XRD) patterns were obtained by θ–2θ scan using an X’Pert diffractometer (Malvern PANalytical B.V., Almelo, Netherlands) equipped with a CuK_α_ radiation source. In order to investigate the in-plane orientations of NbTi alloy thin films, an XRD pattern was obtained by φ scan using a SmartLab diffractometer (Rigaku Corp., Tokyo, Japan). The structure at the interface between the NbTi alloy thin film and STO substrate was observed by STEM (JEM-ARM 200 F, JEOL Ltd., Tokyo, Japan). Samples for the STEM were processed by focused ion beam equipment. The accelerating voltage in observation of the STEM images was 120 kV. The surface morphologies of the thin films were observed in the range of 1 μm × 1 μm by AFM (SPM-9700, Shimadzu Corp., Kyoto, Japan). The superconducting properties of the thin films were evaluated by measuring the temperature dependence of resistance by a four-terminal measurement method using a physical property measurement system (PPMS; Quantum Design Inc., San Diego, CA, USA).

## Electronic supplementary material

Supplementary Information
