# Supplementary material for: Growth and superconductivity of niobium titanium alloy thin films on strontium titanate (001) single-crystal substrates for superconducting joints
Source: Sci Rep. 2018 Oct 11;8:15135. doi: 10.1038/s41598-018-33442-7 (PMC6181932; doi:10.1038/s41598-018-33442-7)
Supplement: Supplementary file 1 — Supplementary Information [file 41598_2018_33442_MOESM1_ESM.pdf]

Supplementary Information

Growth and superconductivity of niobium titanium alloy thin films on strontium titanate (001) single-crystal substrates for superconducting joints

Yuhei Shimizu[1,2,*], Kazuhiko Tonooka[1], Yoshiyuki Yoshida[1], Mitsuho Furuse[1], and Hiroshi Takashima[1,*]

[1]*National Institute of Advanced Industrial Science and Technology (AIST), Central-2, 1-1-1 Umezono, Tsukuba, Ibaraki 305-8568, Japan*
[2]*Present address: National Metrology Institute of Japan (NMIJ), National Institute of Advanced Industrial Science and Technology (AIST), Central-3, 1-1-1 Umezono, Tsukuba, Ibaraki 305-8563, Japan*

*Corresponding authors
Yuhei Shimizu (yuhei-shimizu@aist.go.jp), Hiroshi Takashima (h-takashima@aist.go.jp)

Williamson-Hall plot

By the Williamson-Hall method, the contribution of strain and crystal size can be separated using X-ray diffraction (XRD) pattern data according to the following relation[S1,S2]:

$$\beta \cos\theta = \frac{0.9\lambda}{D} + 4\varepsilon \sin\theta$$

Where θ is the diffraction angle, β is the full width at half maximum of the XRD peak, λ is the wavelength of the X-ray, D is the crystallite size, and ε is the strain. In the relation of β cosθ versus sinθ, the slope and intercept represent the contribution of strain and crystallite size, respectively. Fig. S1 shows the Williamson-Hall plot (β cosθ versus sinθ). It might not be sufficient because there are only two data for each sample, namely 110 and 220 peaks. However, we would be able to understand a trend roughly. When film thickness increase from 10 nm to 50 nm, the slope changed drastically and became gentle. This means that the strain decreased with increasing the film thickness.

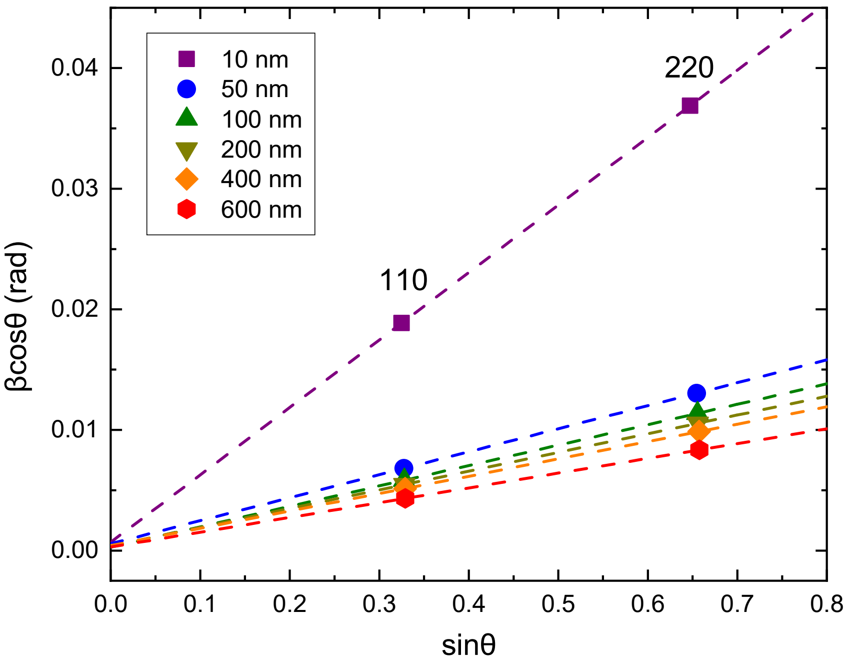

Fig. S1 Williamson-Hall plot of the NbTi alloy thin films with the thickness from 10 nm to 600 nm.

References

S1. Williamson, G. K. & Hall, W. H. X-ray line broadening from filed aluminium and wolfram. *Acta Metall.* **1**, 22-31 (1953).

S2. Choudhury, N. & Sarma, B. K. Structural analysis of chemically deposited nanocrystalline PbS films. *Thin Solid Films* **519**, 2132-2134 (2011).
